# Antioxidant and Antigenotoxic Potential of *Infundibulicybe geotropa* Mushroom Collected from Northwestern Turkey

**DOI:** 10.1155/2020/5620484

**Published:** 2020-02-19

**Authors:** Mustafa Sevindik, Hasan Akgul, Zeliha Selamoglu, Nady Braidy

**Affiliations:** ^1^Department of Food Processing, Bahçe Vocational School, Osmaniye Korkut Ata University, Osmaniye, Turkey; ^2^Department of Biology, Faculty of Science, Akdeniz University, Antalya, Turkey; ^3^Department of Medical Biology, Faculty of Medicine, Ömer Halisdemir University, Nigde, Turkey; ^4^Centre for Healthy Brain Ageing, School of Psychiatry, University of New South Wales, Sydney, Australia

## Abstract

*Infundibulicybe geotropa* (Bull.) Harmaja is an edible mushroom found in Bolu province in northwestern Turkey. The chemical composition and bioactivity of these mushrooms has not been previously investigated. We examined the phenolic composition, elemental content, and antioxidant and antigenotoxic effects of methanol extracts of fruiting bodies. The phenolic compounds in the fungal samples were determined using high-performance liquid chromatography (HPLC), and element content was determined using atomic absorption spectrophotometry. Total antioxidant status (TAS), total oxidant status (TOS), and oxidative stress index (OSI) were determined using the commercially available Rel assay kit. The antigenotoxic effects of the extract were determined using the MTT assay to assess cell viability and the alkaline single-cell gel electrophoresis assay (Comet assay). The total phenolic content (ppm) of *I. geotropa* was found to be catechin (361 ± 2.31), clorogenic acid (553.54 ± 5.06), and coumaric acid (9.93 ± 0.25). The TAS, TOS, and OSI of the extract were 1.854 ± 0.051 mmol/L, 30.385 ± 0.399 *μ*mol/L, and 1.639 ± 0.067, respectively. The elemental levels were within “normal” range. In HT22 mouse hippocampal neuronal cells, the extract (100 and 200 *μ*g/ml) showed no genotoxic potential and ameliorated hydrogen peroxide- (H_2_O_2_-) induced oxidative DNA damage. *I. geotropa* may be considered a good nutrient due to its phenolic constituents and antioxidant potential.

## 1. Introduction

In recent years, the search for natural sources of functional nutrients as a food supplement has become common. Thus, it is quite important to investigate mushrooms, as a natural source of nutrients which have demonstrated potent antioxidant, anti-inflammatory, antitumoral, immunomodulatory, cardioprotective, hepatoprotective, and neuroprotective properties [1–5]. Since ancient times, mushrooms have been used for nutritional and medical purposes and are prevalent in several regions of the world [6–8]. Apart from their attractive taste and culinary uses, edible mushrooms are of nutritional significance owing to their high protein, carbohydrate, vitamin, mineral and phenolic levels, and low-fat content [9–12]. These constituents have been attributed to the biological activities reported for edible mushrooms.

Increased production of free radicals in living organisms and insufficiency of endogenous antioxidants against this increase can result in deterioration of several molecular structures, including lipid, proteins, and nucleic acids. Metabolic and chronic diseases such as cardiovascular disorders, diabetes, cancer, cataracts, muscle degeneration, and neurological diseases such as Parkinson's disease and Alzheimer's disease, are associated with an imbalance between oxidative stress formation and endogenous antioxidant defense mechanisms [13–18]. A growing body of evidence from epidemiological studies and clinical trials has shown that supplementation with exogenous antioxidants and high consumption of antioxidant-rich foods may slow down or delay the onset and progression of many chronic age-related diseases [19, 20].

The beneficial effects of edible mushrooms have been previously investigated. Mushrooms have been shown to scavenge reactive oxygen and reactive nitrogen species, chelate redox-active metals, inhibition lipid peroxidation, and protein carbonyl formation, increase the levels of endogenous antioxidants such as vitamin C and glutathione, and increase the activity of antioxidant enzymes such as glutathione reductase and catalase [21–23]. Several edible mushroom extracts have been shown to protect against DNA damage following exposure to various genotoxicants (H_2_O_2_ radicals, methyl methanesulfonate, cyclophosphamide, and 2-amino-3-methylimidazo(4,5-f)quinolone) in several cell lines, including Burkitt's lymphoma cells, Chinese hamster lung fibroblastic V79 cells, human laryngeal epidermoid carcinoma HEp2 cells, human hepatoma HepG2 cells, and human lymphocytes [24, 25]. Moreover, several bioactive compounds present in mushrooms, which were discovered in molecular studies, have been used in the development of pharmacological agents in recent years [26]. Edible mushrooms play a role in the decomposition of organic materials in nature and contain different levels of elements based on the substrate content they utilize [27]. Mushrooms may possess toxic or nutritional properties based on their elemental composition. Thus, it is important to determine the elemental content of mushrooms consumed as nutrients or medicinal material.

The genus *Infundibulicybe* contains 19 widespread species that are mostly edible. They grow saprotrophic on litter of leaves and needles or can be found in soils in forests, grassland, and alpine habitats. Unlike some other edible mushrooms, *Infundibulicybe* do not have a distinct or foul smell [28]. However, little is known regarding the constituents and biological activity of these mushrooms. In the present study, we aimed to determine the phenolic compounds present in *Infundibulicybe geotropa* (Bull.) Harmaja mushroom. We also assessed its antioxidant and antigenotoxic capacity *in vitro.* We also examined the heavy metal content of these mushrooms to evaluate the nutritional value of these mushrooms as a supplementary nutrient source in humans.

## 2. Materials and Methods

### 2.1. Mushroom Species

*Infundibulicybe geotropa* samples were collected in Bolu province (Turkey). Morphological and ecological characteristics of the samples were noted and photographed in their natural habitats. After field studies, specimen were taken to the laboratory and confirmation of the mushrooms were made by mycological experts at Akdeniz University, Turkey. Micromorphological characteristics were observed by light microscopy using 3% KOH, Congo Red, and distilled water. The names of taxa and authors are quoted according to MycoBank (http://www.mycobank.org/) and Index Fungorum (http://www.indexfungorum.org/).

### 2.2. Extraction

Mushroom samples obtained during field studies were identified and dried at +40°C (Profilo, PFD1350W, Turkey). Then, the samples were pulverized. Afterwards, 10 g of the pulverized samples was macerated (24 h), with 200 mL EtOH (ethanol), MeOH (methanol), and DCM (dichloromethane) using a magnetic stirrer (Gerhardt EV 14). The extracts obtained with the extraction process were condensed via filtration at 40°C with a rotary evaporator to prepare the samples for the experiments (Heidolph Laborota 4000 Rotary Evaporator). The extracts were stored at +4°C until the experiments were conducted.

### 2.3. Determination of TAS, TOS, and OSI

*I. geotropa* mushroom methanol extracts were analyzed using the Rel assay commercial assay kit (Assay Kit Rel Diagnostics, Turkey) to determine the TAS, TOS, and OSI values. Trolox was used as the calibrator in the TAS analysis, and hydrogen peroxide was used as the calibrator in the TOS studies. To determine the OSI, the mmol unit of TAS and the *μ*mol unit of the TOS were cross-converted, and the index value was expressed as percentage [29, 30].

#### 2.3.1. TAS Assay Tests

The TAS assay kit contained Reagent 1 (Buffer), Reagent 2 (Color ABTS Radical Solution), Standard 1 (1.00 mmol trolex Equiv./L), and Standard 2 (1.00 mmol trolex Equiv./L). 200 *μ*L of Reagent 1 was added to the wells on the 96-well plate. 12 *μ*L of mushroom extract was placed on top. The absorbance was measured at 660 nm. Then, 30 *μ*L of Reagent 2 was added and incubated at 37°C during 5 minutes. After incubation, the second absorbance was read at 660 nm. Standard 1 and Standard 2 included in the kit were measured in the same way. Processes for all mushroom extracts were repeated separately.

#### 2.3.2. TOS Assay Tests

The TOS assay kit contained Reagent 1 (Assay buffer), Reagent 2 (Prochromogen solution), Standard 1 (Blank solution: distilled water), and Standard 2 (stock stabilized standard solution (SSSS): 800 mM H_2_O_2_ Equiv./L). For this dilution step, 5 *μ*L of the Standard 2 was transferred to the Eppendorf and 1 mL of distilled water was added and vortexed. Then, 5 *μ*L of this solution was placed into the Eppendorf and 1 ml of water was added and 20 mM H_2_O_2_ was prepared. This solution was reprepared each time. Then, 200 *μ*L of Reagent 1 was first placed in the well on the Eliza plate and 30 *μ*L of mushroom extract was added. The first absorbance was then read at 530 nm (first absorbance of the sample). After the measurement, 10 *μ*L of Reagent 2 was added. It was then incubated for 5 min at 37°C and the second absorbance at 530 nm was read (second absorbance of the sample). The same procedure was repeated for Standard 2. Separate procedures were repeated for all mushroom samples [30].

### 2.4. Determination of Phenolic Compounds

Phenolic compounds in methanol extracts of *I. geotropa* samples were determined using a SHIMADZU system HPLC device and a DAD detector [31]. The injection volume was set up as 20 *μ*L. Mobile phase A: 3% acetic acid and B: methanol was used, and the flow rate was adjusted to 0.8 mL per minute. Chromatographic separation was carried out with an Agilent Eclipse XDB-C18 column (250 × 4.6 mm id 5 *μ*m) at 30°C.

### 2.5. Determination of the Element Content

*I. geotropa* mushroom samples were pulverized after the process of drying. One gram of dried sample was weighed and placed in 50 mL glass vials in 3 repeats. 10 mL concentrated HNO_3_ was added and left at room temperature for 24 h. The samples were heated on the heating plate until the formation of sediment on the surface. Then, 10 mL concentrated HCI was added to these flasks and the incineration process was repeated. Following the incineration, 20 mL diluted HCl was added to the samples and the product was filtered. The elemental content in the filtered samples was then determined with a PerkinElmer (AAS 400) atomic absorption spectrophotometer as previously described [32].

### 2.6. Cell Culture

HT22 mouse hippocampal neuronal cells (BNCC, 337709) were cultured in a cell culture flask at a density of 1 × 10^5^ cells/mL and grown in Dulbecco's modified Eagle's medium (DMEM) supplemented with 10% fetal calf serum (FCS), 1% glutamine, and 1% antibiotic/antifungal in an atmosphere containing 5% CO_2_ and 95% oxygen. All cell culture equipment were obtained from Invitrogen (Melbourne, Australia).

### 2.7. Cell Viability Assay

MTT assay was used to evaluate cell viability following exposure to mushroom methanol extracts as previously described [33]. Briefly, HT22 cells were seeded in 96-well microtiter plates at 1.0 × 10^4^ cells/well. Stock solutions of compounds were prepared in 20 mM DMSO and diluted further in complete DMEM so that the final DMSO concentration was <0.5% (*v*/*v*). After 24 h, the cells were incubated with 50 *μ*l of extracts at a range of concentrations (50–300 *μ*g/ml) for 24 h/37°C in a humidified atmosphere containing 5% CO_2_ and 95% air. After this incubation, 20 *μ*L of MTT (5 mg/mL) was added to each well and further incubated for 4 h at 37°C. The cells were dissolved in 100 *μ*L of DMSO, and the plates were read at 570 nm using a scanning multiwell spectrophotometer. The validity of the MTT assay was confirmed by viable cell counts using Trypan blue [34]. The results were expressed as % viability.

### 2.8. Antigenotoxicity Assay

The antigenotoxicity/genotoxicity of methanol extracts of edible mushrooms was evaluated using the alkaline single-cell gel electrophoresis (Comet) assay as previously described [35]. Briefly, distilled water was used as negative control, while H_2_O_2_ (100 *μ*M) was the positive control. After incubation with mushroom extract (100 and 200 *μ*g/ml) for 24 h, cells were de-attached by trypsinisation for the Comet assay. For each treatment, the cell suspension were mixed with low-melting point agarose, of which 90 *μ*L was added to 15 *μ*L of the cell homogenate and placed on a microscope slide precoated with normal melting point agarose 1.0%. A coverslip was added, and the slides were placed on ice during 5 min. After solidification, the coverslips were removed and the slides were immersed in a lysis solution. The slides were kept frozen in the lysis solution (4°C) and protected from light for approximately 14 h. They were subsequently incubated in freshly prepared alkaline buffer for 20 min for DNA unwinding. Electrophoresis (20 min at 300 mA and 25 V) was carried out in the same buffer. The procedure was performed under dimmed light to prevent further DNA damage. After electrophoresis, the slides were rinsed with distilled water and neutralized in Tris 400 mM (pH 7.5) and left to dry overnight at room temperature. The dry slides were rehydrated for 3 min in distilled water and then fixed for 10 min, rinsed three times in distilled water, and dried for at least 5 h. The dry slides were rehydrated for 3 min in distilled water, stained, and constantly shaken for 25 min. The slides submerged in the stop solution were rinsed again and immediately tagged for analysis. Comets were visualized using ethidium bromide staining (20 *μ*g/ml for 30 s) and a fluorescent microscope (Nikon Eclipse 600, Japan). The slides were analyzed under blind conditions by at least two individuals. DNA damage was given as DNA damage index (DI). The OpenComet plugin was used for comet scoring. The results were reported as % tail DNA.

### 2.9. Statistical Analysis

All experiments were performed 5 times, and results expressed as mean ± standard deviation unless otherwise stated. Statistical analysis using Student's *t* test was performed using Microsoft Excel. Results were considered significant when *p* < 0.05.

## 3. Results and Discussion

### 3.1. Phenolic Content of *I. geotropa*

In this study, the phenolic compounds in *I. geotropa* mushroom ethanol extracts were determined using HPLC, and the findings are presented in Table 1. Analysis of phenolic compounds demonstrated that there are 4 main phenolic substances in *I. geotropa*: 361.49 ± 2.31 ppm catechin; 553.54 ± 5.06 ppm chlorogenic acid, and 9.93 ± 0.25 ppm coumaric acid. It is well-established that catechin is a potent antioxidant compound that is involved in the inhibition of free radicals. It has also been shown to have protective effects against skin, breast, prostate, and lung cancers in addition to its antioxidant properties [36]. Chlorogenic acid was reported to possess several biological activities such as antioxidant and anti-inflammatory effects, as well as regulation of glucose and lipid metabolisms, antidiabetic, anticarcinogenic, anti-inflammatory, and antiobesity effects [37]. Coumaric acid was shown to have several biological activities including antioxidant, anticancer, antimicrobial, antiviral, anti-inflammatory, antithrombocyte aggregation, anxiolytic, antipyretic, analgesic, and mitigating effects on diabetes, obesity, hyperlipemia, and gout [38]. Another study of phenolic content conducted in *I. geotropa* (former name *Clitocybe geotrapa*) showed that, apart from the named phenolic compounds identified in this study, protocatechuic acid, p-hydoxybenzoic acid, absisic acid, and cinnamic acid may also be present in the mushroom [39]. Therefore, given their phenolic content, *I. geotropa* may be consumed as a natural source for antioxidant phenols and may exert important health benefits.

### 3.2. TAS, TOS, and OSI Values for *I. geotropa*

Mushrooms have the potential to contain numerous antioxidant enzymes and reduced coenzymes such as phenolic compounds as an effective electron source in several different forms and types. Furthermore, they are rich in antioxidant vitamins A, C, and E, and other natural products that could metabolically contain and produce several elements with redox potential and strong antioxidant character. Previous studies on *I. geotropa* did not report the oxidative stress status of the mushroom species. Limited information is available in the literature on the TAS, TOS, and OSI values in other edible mushrooms. In the present study, TAS (mmol/L), TOS (*μ*mol/L), and OSI values were determined using the *I. geotropa* ethanol extract. The findings are presented in Table 2.

As shown in Table 2, the TAS value of the mushroom ethanol extract was 1.854 ± 0.051 mmol/L. Studies that aimed to determine these values demonstrated that the TAS values in *Tricholoma terreum*, *Coprinus micaceus*, *Pleurotus eryngii*, *Auricularia polytricha*, *A. auricular*, *Omphalotus olearius*, *Macrolepiota procera*, *Trametes versicolor*, *Geastrum pectinatum*, and *Fomitopsis pinicola* were 0.38, 0.46, 1.93, 0.93, 1.010, 2.836, 2.823, 0.820, 1.278, and 1.57 mmol/L, respectively [40, 41]. The reported TAS value for *I. geotropa* in our study was significantly lower compared to the reported values for *P. eryngii, O. olearius*, and *M. procera* mushrooms as determined in previous studies. We also found that the mushroom had a higher TAS value when compared to the other abovementioned mushrooms. These differences may be attributed to the count and types of phenolic constituents, the release of secondary minerals produced by endogenous and exogenous factors, variation in antioxidant vitamin levels, as well as changes in the level of enzymatic/nonenzymatic antioxidant molecules capable of altering the total antioxidant capacity.

Our study also reported that the TOS value of the mushroom ethanol extract was 30.385 ± 0.399 *μ*mol/L. Previous reports on mushroom TOS values demonstrated that the TOS values of *T. terreum*, *C. micaceus*, *O. olearius*, *M. procera*, *A. auricular*, and *T. versicolor* were 16.76, 16.87, 8.26, 10.35, 23.91, and 2.03 *μ*mol/L, respectively [40, 41]. Our current study demonstrates that the TOS value of *I. geotropa* was generally much higher when compared to the findings obtained in previous studies. Differences between mushroom TOS values were noteworthy since the analyses were carried out by different research groups at different times and with different species of mushroom collected in different locations. The differences among the TOS values are likely due to the differences between the regions where they were collected and the impact of the differences between the metabolic processes of the different mushroom species on their capacity to produce and accumulate oxidant compounds. It is suggested that mushrooms or other naturally occurring products with high TOS values should be cautiously consumed. It is considered that high TOS values in plants may be due to the impact of environmental and metabolic factors, which may stimulate the production of free radicals to protect against harmful endogenous factors in the environment such as environmental pollutants and certain microorganisms and parasites. Therefore, our findings highlight the importance of collecting mushrooms to be used as nutrients or in drug design studies in areas that are free of environmental toxic agents and heavy metal pollution and to enhance public awareness of those who work in the field.

The OSI value reflects the rate of inhibition of the oxidant compounds produced by mushrooms due to environmental and/or inherent factors by the antioxidant compounds present in the organism. Previous studies on the determination of OSI values reported that the OSI was 4.41, 3.67, 0.29, 0.37, 2.37, 2.17, 1.08, and 0.13 for *T. terreum*, *C. micaceus*, *O. olearius*, *M. procera*, *A. auricula*, *T. versicolor*, *G. pectinatum*, and *F. pinicola*, respectively [40, 41]. The OSI for *I. geotropa* (1.639 ± 0.067) in our study was observed to be lower than that of *T. terreum*, *C. micaceus*, *A. auricula*, and *T. versicolor* mushrooms. The higher TOS values observed in the present study coupled with a higher TAS in the mushroom samples led to the observation of a lower OSI value. Oxidative stress induced by oxidant molecules was prevented by TAS, which covers the whole enzymatic and nonenzymatic systems, resulting in low OSI values.

### 3.3. Elemental Content of *I. geotropa*

Elements also play a role as cofactors in the functioning of certain enzymes, which are a part of several metabolic pathways and the endogenous antioxidant system. Given the cofactor properties of the elements and their potential effects on homeostasis (protection of the internal balance of the body), the identification of elemental content of mushrooms is of great importance. Mushrooms physiologically accumulate elements at different levels based on the elemental content of the substrate they utilize. In the present study, the elemental content of *I. geotropa* was determined and presented in Table 3.

The Fe content in *I. geotropa* was 63.70 ± 8.88, while the Zn content was 61.24 ± 12.04, Cu content was 30.37 ± 1.23, Pb content was 7.00 ± 1.38 and Ni content was 1.12 ± 0.053 mg·kg^−1^. A previous study reported that the Fe content in *I. geotropa* mushroom collected in Muğla province (Turkey) was 662.0, Zn content was 130.4, Cu content was 65.6, Pb content was 3.2, and Ni content was 4.5 mg·kg^−1^ [42]. It was determined that the Fe content in *I. geotropa* mushroom collected in Eskişehir province (Turkey) was 516.7, Zn content was 86.6, Cu content was 82.4, Pb content was 1.22, and Ni content was 14.0 mg·kg^−1^ [43]. Comparatively, it was found that Fe, Zn, Cu, and Ni contents in *I. geotropa* collected in Bolu (Turkey) were lower. However, the Pb content was higher in the present study when compared to both abovementioned studies. The difference in the level of these elements stems from variation in the elemental soil content between the regions where the mushrooms were collected. Furthermore, it is considered that the high Pb content may be since the area where the mushrooms were collected in the present study was located near a busy highway.

Fe is a very important element that is necessary in different amounts in different periods for the human body. Fe deficit leads to several diseases, especially anemia (Ibrahim et al., 2012). Fe requirement is met by breast milk for the 6 postnatal months. Approximately 0.7-0.9 mg/day of Fe is utilized after 6 months (FAO/WHO, 2004). Between the ages of 1 and 6, the body needs twice as much iron and 1.17 mg/day Fe is needed between the ages of 6 and 15. Furthermore, male individuals aged 12-16 years require 1.82 mg/day of iron, while female individuals require 2.02 mg/day. During pregnancy, the Fe requirement is 1.14 mg/day, and an adult female needs approximately 2 mg/day of iron during menstruation and premenopausal period [44]. In the present study, 63.70 mg Fe was found in 1 kg *I. geotropa*. This amount is much higher than an individual's daily iron requirement. Thus, it is considered that the consumption of the mushroom samples collected in suitable regions at certain intervals may be beneficial in meeting the physical Fe requirement, and the findings of the present study would be beneficial in making recommendations for the regular consumption of mushrooms collected in reliable localities.

Ni content is lower than 0.5 mg·kg^−1^ in most food products. However, cocoa products and peanuts may contain up to 3 to 10 mg·kg^−1^ Ni [45]. It can be argued that the Ni content determined in 1 kg of *I. geotropa* mushroom is 1.12 mg and did not reach toxic levels. On the other hand, it was determined that the lowest and highest mushroom Pb content was 2.86-15.3 while the Zn and Cu contents were 29.8-158 and 6-187 mg·kg^−1^, respectively, in previous studies by our group [40, 42, 46]. Compared to these values, the Pb, Zn, and Cu content determined in the *I. geotropa* appeared to be within normal ranges identified in the literature. Thus, it was determined that the *I. geotropa* samples collected in Bolu (Turkey) possessed optimal element levels, and consumption of the mushroom is adequate for human health. However, controlled consumption and avoiding consumption at extreme levels are recommended.

### 3.4. Antigenotoxic/Genotoxic Effects of *I. geotropa*

**Figure 1:**
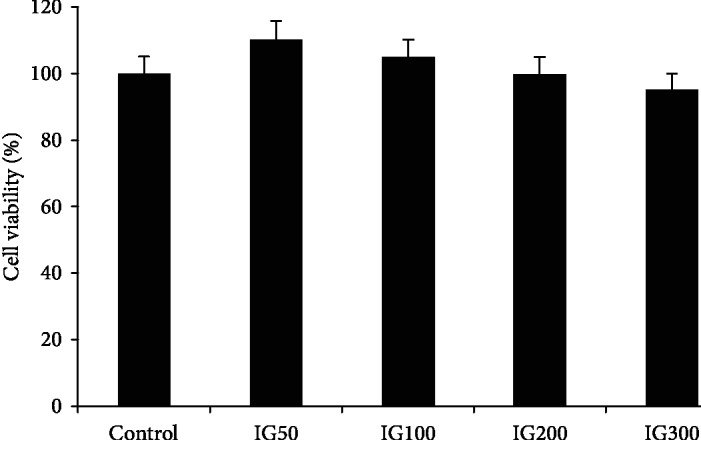
Viability of HT22 cells after 24 h treatment with *Infundibulicybe geotropa extract* (50, 100, 200, and 300 *μ*g/ml). Results are presented as mean ± SE of 5 independent experiments. ^∗^*p* < 0.05 when compared with control (*t* test).

**Figure 2:**
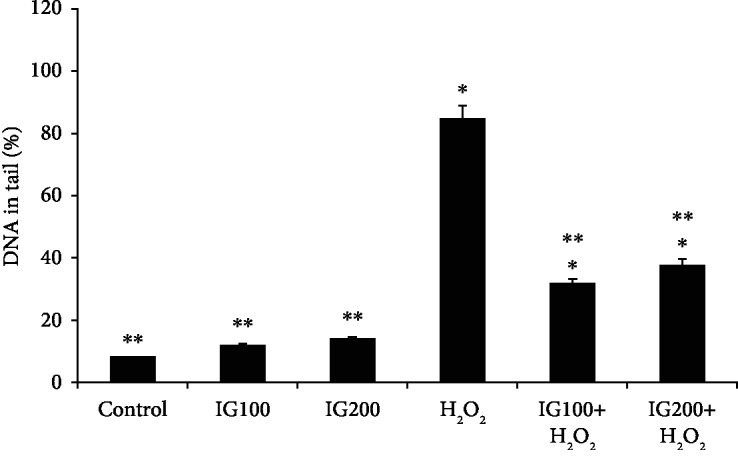
Percentage tail DNA in HT22 cells after 24 h pretreatment with 100 and 200 *μ*g/ml *Infundibulicybe geotropa extract*. Results are presented as mean ± SE of 5 independent experiments. ^∗^*p* < 0.05 when compared with control (*t* test); ^∗∗^*p* < 0.05 when compared with H_2_O_2_ treatment alone (*t* test).

To the best of our knowledge, there are no previous reports of the toxicity of *I. geotropa* extract in neuronal cells. Our study investigated the effects of *I. geotropa* in HT22 cells. Our data shows that *I. geotropa* extracts did not significantly reduce cell viability at 50-300 *μ*g/ml after 24 h (Figure 1). Other mushroom extracts, e.g., *A. blazei*, *G. Frondosa*, and *H. erinaceus*, were found to be toxic to Chinese hamster fibroblast cells after 24 h at concentrations of 2 mg/ml [47]. We also examined the ability of *I. geotropa* to protect against oxidative DNA damage using the Comet assay (Figure 2). This versatile assay allows the detection of single- and/or double-strand DNA breaks using H_2_O_2_ as a genotoxic agent. Our data shows that H_2_O_2_ induced a significant increase in oxidative DNA damage (as indicated by the Comet tail) compared to nontreated control cells (Comet head only). After 24 h, treatment with *I. geotropa* extract (100 and 200 *μ*g/ml) ameliorated the increase in DNA damage due to H_2_O_2_. The genoprotective effect of *I. geotropa* may be attributed to its phenolic content and high TAS levels. We hypothesize that *I. geotropa* extract may exert protection against H_2_O_2_ by either direct scavenging of free radicals and/or upregulation of endogenous antioxidant enzymes (e.g., catalase, glutathione peroxidases) and antioxidant concentrations (e.g., glutathione) which detoxify H_2_O_2_. Other mushroom extracts (e.g., *A. bisporus* and *G. lucidum*) have been shown to protect against induced DNA damage in Raji cells [25]. Moreover, the antigenotoxic effects of *A. bisporus* extracts have also been attributed to enhanced tyrosinase activity, which can enhance endogenous antioxidant defense mechanisms leading to increase glutathione levels [48].

While most studies have shown that edible mushrooms are safe and well tolerated, some studies have shown that they may exhibit some genotoxic potential. For example, extracts of *A. blazei*, was shown to be genotoxic to HTC rat hepatoma cells. However, this effect was likely due to the generation of cytotoxic metabolites *in vitro* [49]. The genotoxic potential of mushrooms is essential to accurately determine the edibility and safety profile of mushrooms for human consumption. The limited toxicity and significant antigenotoxic effects of *I. geotropa* extracts in murine HT22 neuronal cells is encouraging and warrants further investigation to elucidate the modes of action underlying their antigenotoxic effects.

## 4. Conclusion

Mushrooms provide significant opportunities in the fields of pharmacological agent development, drug design, and medicine due to their structural properties and exhibit various metabolic activities in addition to their nutritional properties. In this study, the biological activity in *Infundibulicybe geotropa* mushroom collected in Bolu province (Turkey) was determined. Catechin, chlorogenic acid, and coumaric acid were determined in the mushroom in the present study. It was considered that this mushroom species may be a natural resource, rich in the determined compounds. It was also found that the mushroom has a strong antioxidant potential. However, it has been suggested that fungi samples collected in more reliable regions containing lower oxidant levels may serve as a better antioxidant source. The extract also showed excellent protection against oxidative DNA damage and lacks genotoxic potential *in vitro*. The elemental content was at optimal levels as well, suggesting that this extract may be used as an antioxidant with little or no adverse effects. However, additional *in vivo* studies are necessary to confirm these assumptions.

## Figures and Tables

**Table 1:** Phenolic contents of mushroom.

Phenolic compound (ppm)	Catechin	Chlorogenic acid	Coumaric acid
*I. geotropa*	361.49 ± 2.31	553.54 ± 5.06	9.93 ± 0.25

**Table 2:** TAS, TOS, and OSI values of mushroom.

	TAS (mmol/L)	TOS (*μ*mol/L)	OSI
*I. geotropa*	1.854 ± 0.051	30.385 ± 0.399	1.639 ± 0.067

**Table 3:** Element contents of mushroom.

	Fe (mg·kg^−1^)	Zn (mg·kg^−1^)	Cu (mg·kg^−1^)	Pb (mg·kg^−1^)	Ni (mg·kg^−1^)
*I. geotropa*	63.70 ± 8.88	61.24 ± 12.04	30.37 ± 1.23	7.00 ± 1.38	1.12 ± 0.053

## Data Availability

All molecular data used to support the findings of this study are available from the corresponding author upon request.
